# Elucidating the Regulon of a Fur*-*like Protein in *Mycobacterium avium* subsp. *paratuberculosis* (*MAP*)

**DOI:** 10.3389/fmicb.2020.00598

**Published:** 2020-04-23

**Authors:** Fernanda Miyagaki Shoyama, Taveesak Janetanakit, John P. Bannantine, Raul G. Barletta, Srinand Sreevatsan

**Affiliations:** ^1^Department of Pathobiology and Diagnostic Investigation, College of Veterinary Medicine, Michigan State University, East Lansing, MI, United States; ^2^Department of Veterinary Public Health, Faculty of Veterinary Science, Chulalongkorn University, Bangkok, Thailand; ^3^National Animal Disease Center, USDA-Agricultural Research Service, Ames, IA, United States; ^4^School of Veterinary Medicine and Biomedical Sciences, University of Nebraska, Lincoln, NE, United States

**Keywords:** *Mycobacterium avium* subsp. *paratuberculosis*, Fur, iron, regulon, ChIP-seq

## Abstract

Intracellular iron concentration is tightly regulated to maintain cell viability. Iron plays important roles in electron transport, nucleic acid synthesis, and oxidative stress. A *Mycobacterium avium* subsp. *paratuberculosis* (*MAP*)-specific genomic island carries a putative metal transport operon that includes *MAP3773c*, which encodes a Fur-like protein. Although well characterized as a global regulator of iron homeostasis in multiple bacteria, the function of Fur (ferric uptake regulator) in *MAP* is unknown as this organism also carries IdeR (iron dependent regulator), a native iron regulatory protein specific to mycobacteria. Computational analysis using PRODORIC identified 23 different pathways involved in respiration, metabolism, and virulence that were likely regulated by *MAP3773c*. Thus, chromatin immunoprecipitation followed by high-throughput sequencing (ChIP-seq) was performed to confirm the putative regulon of *MAP3773c* (Fur-like protein) in *MAP*. ChIP-Seq revealed enriched binding to 58 regions by Fur under iron-replete and -deplete conditions, located mostly within open reading frames (ORFs). Three ChIP peaks were identified in genes that are directly related to iron regulation: *MAP3638c* (hemophore-like protein), *MAP3736c* (Fur box), and *MAP3776c* (ABC transporter). Fur box consensus sequence was identified, and binding specificity and dependence on Mn^2+^ availability was confirmed by a chemiluminescent electrophoresis mobility shift assay (EMSA). The results confirmed that *MAP3773c* is a Fur ortholog that recognizes a 19 bp DNA sequence motif (Fur box) and it is involved in metal homeostasis. This work provides a regulatory network of *MAP* Fur binding sites during iron-replete and -deplete conditions, highlighting unique properties of Fur regulon in *MAP.*

## Introduction

*Mycobacterium avium* subsp. *paratuberculosis* (*MAP*) is the causative agent of Johne’s disease (JD) in ruminants, a chronic and incurable chronic enteritis characterized by persistent diarrhea that leads to malnutrition and muscular wasting (Rathnaiah et al., 2017). JD is present worldwide and imposes significant economic losses to the dairy industry (Garcia and Shalloo, 2015). Unfortunately, to date, reliable JD diagnostics are still lacking. Culture of *MAP* from feces has been the most reliable method for diagnosis of JD; however, *MAP* requires 8 to 16 weeks to produce colonies in culture, presenting a major hurdle to diagnosis (Bannantine et al., 2002).

Unlike other mycobacteria, *MAP* has special iron requirements. For optimal growth *in vitro*, it requires supplementation of the siderophore mycobactin J. Whole-genome sequencing of *MAP* K-10 provided a potential explanation for this dependency, revealing a truncation of the *mbtA* gene, with *MAP* making a protein that is 151–156 amino acids shorter than *M. tuberculosis* or *M. avium* (Li et al., 2005). It has been suggested that this truncation impairs the production of mycobactin from the *mbtA–J* operon (Li et al., 2005; Wang et al., 2014). Despite this truncation, Zhu et al. (2008) showed that *MAP* is still able to transcribe mycobactin synthesis genes inside macrophages. To corroborate these findings, Janagama et al. described the upregulation of several genes responsible for iron acquisition in infected tissues, including genes responsible for mycobactin biosynthesis (Janagama et al., 2010).

Iron is vital to fundamental biological processes, however, high intracellular concentrations of free iron are toxic to bacteria. As such, cells have developed tightly regulated processes for intracellular metal homeostasis (Eckelt et al., 2014). Bacteria control metal homeostasis by activating a set of genes regulated by metal-sensing transcription factors known as metalloregulatory proteins (Chandrangsu et al., 2017). In prokaryotes, there are two major families of metalloregulators: diphtheria toxin (DtxR) and ferric uptake regulator (Fur) (Hantke, 2001). In 2009, Janagama and others identified and characterized *MAP2827*, an iron-dependent regulator (IdeR) in *MAP*. A member of the DtxR protein family, IdeR is involved in regulatory mechanisms to acquire, store, or prevent excess accumulation of iron. The authors were able to confirm that *MAP2827* was in fact IdeR and regulates genes involved in iron acquisition (*mbtB*) and iron storage (*bfrA*) (Janagama et al., 2009). However, *in vitro* iron stress showed that IdeR regulation is strain dependent, while IdeR from *MAP* cattle strain K-10 regulates mycobactin synthesis and storage genes similar to IdeR from *M. tuberculosis*. IdeR from *MAP* sheep strain S397 shows deficiency in iron storage function, resulting in a strain more sensitive to iron fluctuations (Janagama et al., 2010).

In addition to IdeR, *MAP* genome contains a putative metal transport *MAP*-specific operon and large genomic polymorphisms (LSPs), 15, that include a Fur-like transcriptional regulator, *MAP3773c* (Alexander et al., 2009). First identified in *Escherichia coli*, Fur has been shown to respond to iron-replete conditions to repress gene expression and allow sufficient concentration of intracellular iron for essential iron-related activities (Hantke, 1981; Bagg and Neilands, 1987; Lee and Helmann, 2007). Similar to several representatives of Fur family member, Fur protein requires binding of a divalent metal ion, either Fe^2+^ or Mn^2+^, for DNA-binding activation (Mills and Marletta, 2005; Lee and Helmann, 2007; Chandrangsu et al., 2017). Fur protein generally binds to a 19-bp inverted repeat sequence known as a “Fur box” (GATAATGATwATCATTATC; w = A or T), within the promoter of the regulated genes (Escolar et al., 1999). In *MAP*, functional genomics suggested three Fur boxes located in a 38-kb *MAP*-specific genomic island (LSP14) (Stratmann et al., 2004; Alexander et al., 2009). *MAP* genome includes a total of six specific genomic insertions: LSP4, LSP11, LSP12, LSP14, LSP15, and LSP16 (Alexander et al., 2009). As these islands are not presented in any other mycobacteria, it has been proposed and confirmed that they were acquired via horizontal gene transfer (Alexander et al., 2009; Wang et al., 2016). Furthermore, LSP14 and LSP15 encode several predicted genes involved in metal uptake systems.

To date, there have been characterization of the other Fur family members in *MAP*, FurA, and FurB, also known as Per (peroxidase stress response) and Zur (zinc uptake repressor), respectively, however, no information about the potential roles of Fur-like element has been described (Eckelt et al., 2014, Eckelt et al., 2015).

As a key virulence determinant, iron regulation in *MAP* and its role in pathogen survival and infection are important areas of research that may lead to advances in ability to improve culturing methods. To further elucidate the mechanisms of iron homeostasis in *MAP*, we investigated the putative function of the Fur*-*like gene (*MAP3773c*) in iron homeostasis *in vitro*. We applied *in vivo* ChIP-seq to confirm binding of *MAP* Fur as a transcription factor and to identify the regulon of genes under its control.

## Materials and Methods

### Bacterial Strains

*MAP* K-10 strain was grown at 37°C without shaking in Middlebrook 7H9 supplemented with 10% OADC (oleic acid, dextrose, catalase) enrichment medium (Thermo Fisher Scientific, Waltham, MA, United States), 0.05% Tween 80, and 2 mg of ferric mycobactin J (Allied Monitor Inc., Fayette, MO, United States) per liter. Antibiotics (μg/ml: kanamycin, 20; hygromycin, 100; streptomycin, 20) were added when necessary. Competent *E. coli* BL21(DE3) (EMD Biosciences, Madison WI, United States) and *E. coli* TOP10F cells (Invitrogen, Carlsbad, CA, United States) were grown in LB medium 37°C with shaking at 200 RPM.

### Protein Expression

To express *MAP* Fur protein, competent *E. coli* BL21(DE3) (EMD Biosciences, Madison WI) carrying *MAP3773c* on pET-24b(+) were growing in LB medium with 30 μg/ml kanamycin. Cultures were kept at 37°C with shaking at 200 RPM for 4 h aerobic growth, until OD_600_ of 0.4 was obtained. Then, protein expression was induced with addition of 0.1 M IPTG and shaking at 37°C for an additional 2 h. The expressed *MAP3773c* was extracted using B-PER (Bacterial Protein Extraction Reagent; Pierce Biotechnology, Rockford, IL, United States), followed by purification using HisPur Ni-NTA resin columns per the manufacturer’s protocol (Pierce Biotechnology, Rockford, IL, United States). Purified protein was analyzed by SDS-PAGE and Western Blot using standard methods described previously (Bannantine and Paustian, 2006). The target band identified from the SDS-PAGE was excised for LC-MS/MS at Michigan State University Proteomics Facilities. Raw data were analyzed using Scaffold (Proteome Software, Portland, OR, United States).

### Western Blotting

*MAP* K-10 were cultured as previously described until reaching an OD_600_ of ∼0.5. For iron starvation, cultures were treated with 2,2′-bipyridyl (DIP, 200 μM final) for 2 h shacking at 200 rpm at 37°C. Cells from iron-replete and -deplete conditions were washed with 1 × PBS and resuspended in freshly made buffer lysis buffer (20 mM HEPES; 50 mM KCl; 0.5 mM DTT; 10% glycerol; mini protease inhibitor), followed by cell lysing with MagNA Lyser (Roche Diagnostics, Sandhofer, Germany). For enrichment of Fur protein, samples were subjected to immunoprecipitation. Samples were incubated overnight with antibody for Fur detection at 4°C on a rotating platform followed by 2 h incubation [0.5 h at 4°C and 1.5 h at room temperature (RT)] on a rotating platform. Samples were washed two times with IPP150 buffer (10 mM Tris–HCl; 150 mM NaCl, 0.1% NP40) and two times with 1 × TE (0.05 M Tris–HCl; 10 mM EDTA) buffer. Beads were resuspended in elution buffer and incubated at 65°C for 15 min. The samples were subjected to SDS-PAGE and transferred to Nitrocellulose Membrane, 0.2 μm (Bio-Rad Laboratories, Hercules, CA, United States). Custom-made antibody that binds the *MAP* Fur protein (Genscript, Piscataway, NJ, United States) was used as primary antibody. Anti-rabbit IgG (whole molecule)–peroxidase antibody produced in goat (Sigma-Aldrich, St. Louis, MO, United States) was used as secondary antibody. The membrane was visualized with ChemiDoc MP Imaging System (Bio-Rad Laboratories, Hercules, CA, United States).

### Computational Prediction of Fur-Regulated Genes

Virtual Footprint, part of The Prokaryotic Database of Gene Regulation (PRODORIC) (Münch et al., 2005), was used for prediction of Fur binding site. *MAP* K-10 genome was used as input DNA sequence, Fur box motif from *E. coli* was used as Position Weight Matrix, and searches were limited to -300 to +100 bases of each predicted ORF.

### Chromatin Immunoprecipitation Followed by Sequencing (ChIP-Seq)

ChIP-enriched DNA samples were harvested following the protocol developed by Jaini et al. (2014) using a custom-made antibody that binds the *MAP* Fur protein (Genscript, Piscataway, NJ, United States). *MAP* K-10 culture with an OD_600_ of ∼0.5 was used to generate ChIP-DNA. In order to avoid false positive, input DNA was used as control, and this sample did not have ChIP enrichment. For iron starvation, cultures were treated with DIP (200 μM final) for 2 h shacking at 200 rpm at 37°C. Cells from iron-replete and -deplete conditions were washed with 1 × PBS. Formaldehyde was added at a final concentration of 1% and incubated at RT for 20 min in a platform rocker. Cross-linking was quenched by adding 250 mM of glycine and incubating for 15 min. Cells were washed two times with ice-cold 1 × PBS and resuspended in freshly made buffer lysis buffer (20 mM HEPES; 50 mM KCl; 0.5 mM DTT; 10% glycerol; mini protease inhibitor), followed by cell lysing with MagNA Lyser (Roche Diagnostics, Sandhofer, Germany). Cell suspensions were sonicated using Covaris M220 Focused-ultrasonicator (Covaris, Inc., Woburn, MA) for 18 min; 75.0 peak power; 20.0 duty factor, and 200 cycles/burst. Samples were incubated overnight with antibody for Fur detection at 4°C on a rotating platform followed by 2 h incubation (0.5 h at 4°C and 1.5 h at RT) on a rotating platform. Samples were washed two times with IPP150 buffer (10 mM Tris–HCl; 150 mM NaCl, 0.1% NP40) and two times with 1 × TE (0.05 M Tris–HCl; 10 mM EDTA) buffer. Beads were resuspended in elution buffer and incubated at 65°C for 15 min. 1 mg/ml of Proteinase K was added to each sample and incubated at 37°C for 2 h and transferred for 65°C for overnight incubation. DNA purification was performed using AmPure^xp^ beads per the manufacturer’s protocol (Beckman Coulter, Indianapolis, IN, United States). Sample quality was analyzed by an Agilent 2100 Bioanalyzer (Agilent Technologies, Santa Clara, CA, United States).

### ChIP-Seq Library Construction and Sequencing

DNA fragments ∼300 bp were selected for library preparation and sequencing libraries were prepared using NEXTflex^TM^ ChIP-seq kit (PerkinElmer, Austin, TX, United States) as per the manufacturer’s protocol. Pre- and post-library construction, chromatin immunoprecipitation products were quantified using a Qubit fluorometer (Invitrogen, Carlsbad, CA, United States) and an Agilent 2100 Bioanalyzer (Agilent technologies, Santa Clara, CA, United States). ChIP DNA replicates were pooled and sequenced. Approximately 20M reads per sample were generated, providing 150–1,000 depth of coverage. Sequencing was performed by ACGT, Inc. (Chicago, IL, United States).

### ChIP-Seq Data Analysis

All analysis was done using CLC Genomics Workbench software 12.0 (QIAGEN, Aarhus, Denmark). Raw data generated from ChIP-seq were trimmed and mapped to the reference *MAP* K-10 genome (NCBI accession number NC_002944). Using CLC shape-based peak caller, ChIP-enriched DNA were aligned onto Input DNA (no ChIP enrichment); when the sequence coverage of a genomic region in the enriched DNA exceeded the Input DNA, a ChIP peak score was called. A list of all ChIP peaks with their respective *P* value was generated. The threshold for signal-to-noise ratio (ChIP-enriched DNA vs. no enriched) was set based on false discovery rate (FDR) value equal to or smaller than 10^–50^. FDR was calculated using Bonferroni correction on R software based on the *P* value generated by CLC.

### Motif Detection

A Fur binding motif was generated using Find Individual Motif Occurrences (FIMO), part of the MEME suit (Grant et al., 2011), for all *in vivo* binding sites identified in ChIP-seq analyses. A *P* value of ≤ 0.001 was defined as statistical threshold for Fur binding motifs.

### Electrophoretic Mobility Shift Assay (EMSA)

Physical binding of *MAP3773c* to the promoter sequences of *MAP* Fur box 1 (*MAP3736*c) was carried out by EMSA. Promoter sequences containing Fur box motifs were amplified using 5′ biotin-labeled primer via PCR. Purification of PCR products was done using the QIAquick PCR Purification kit (Qiagen, Germantown, MD, United States). Recombinant *MAP* Fur protein was expressed as stated above. Binding reaction: 1 × Binding Buffer (50 mM Tris–HCl; 25% glycerol; 10 μg/ml Salmon tests DNA; 250 nM NaCl; 5 mM DTT; 250 μg/ml BSA; nuclease free water), 10 mM MnCl_2_, 0–10 nM *MAP* Fur protein, 0–4 pmol Unlabeled DNA, and 20 fmol labeled DNA. The reactions were incubated for 30 min at RT followed by electrophoresis in a 5% native polyacrylamide gel [40% 19:1 Acrylamide; 50% Tris-Acetate (TA) buffer; 50% glycerol; 10% Ammonium persulfate (APS); 6% TEMED] using 1 × TA Buffer (1 M Tris acetate, 0.5 M Glacial acetic acid) as running buffer. After electrophoresis, gels were transferred onto a Biodyne B Nylon membrane (Pierce, Biotechnology, Rockford, IL, United States) and reactions were detected using chemiluminescence-based nucleic acid detection kit (Pierce, Biotechnology, Rockford, IL, United States).

## Results

### Genome-Wide Analysis of Fur Regulon

**FIGURE 1:**
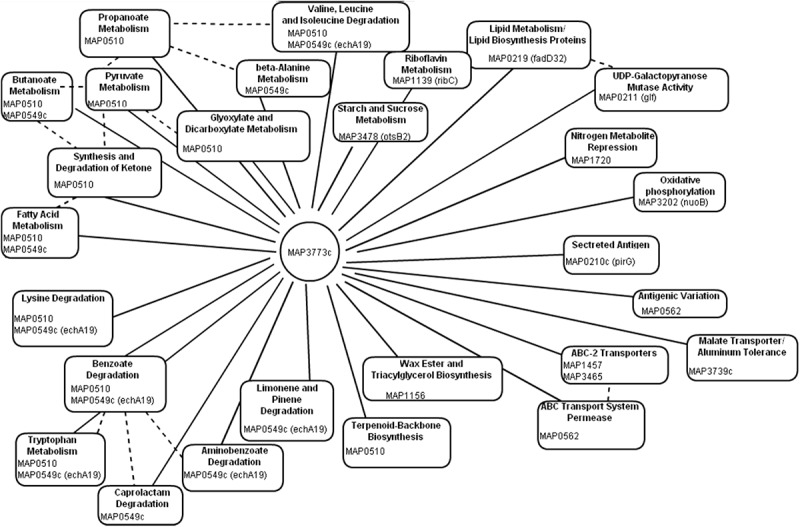
*In silico* analysis of Fur regulon. Using PRODORIC for *MAP* K-10 genome analysis to detect putative Fur binding and predict pathways regulated by *MAP3773c*. Solid lines represent pathways directly regulated by *MAP3773c*. Dashed lines indicated interrelated pathways.

**FIGURE 2:**
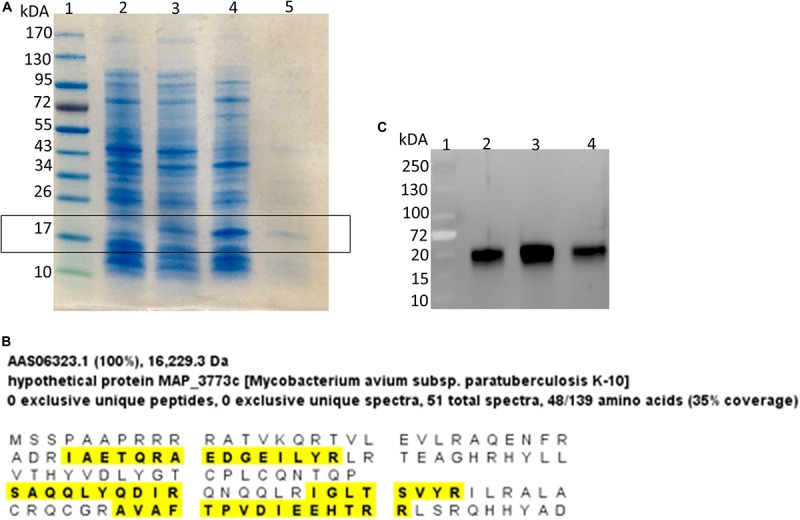
Identification of MAP Fur protein. **(A)** Coomassie stain of SDS-PAGE analysis of Fur expression in *E. coli* system. Lane 1, Protein ladder; Lane 2, BL21(DE3) carrying empty vector pET24b(+); Lane 3, BL21(DE3) carrying *MAP3773c* on pET24b(+); Lane 4, BL21(DE3) carrying MAP3773c on pET24b(+) with addition of IPTG; Lane 5, Purified recombinant *MAP3773c* protein. **(B)** Scaffold analysis of LC-MS/MS data from excised band from lane 5 showing peptide hits (yellow highlights) to 35% of complete *MAP* Fur sequence. **(C)** Western blot showing immunoprecipitation of Fur protein by anti-Fur antibody from *MAP* K-10 cultured under iron-replete and deplete condition. Lane 1, Protein ladder; Lane 2, Pull down of *MAP* K-10 cultured under iron-replete condition using 1 μg of anti-Fur antibody; Lane 3, Pull down of *MAP* K-10 cultured under iron-replete condition using 2 μg of anti-Fur antibody; Lane 4, Pull down of *MAP* K-10 cultured under iron-deplete condition using 1 μg of anti-Fur antibody.

**FIGURE 3:**
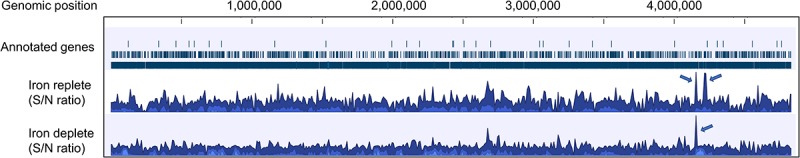
Overview of the mapped sequences within the reference genome *MAP* K-10 under iron-replete and -deplete conditions generated by CLC genomic workbench. After mapping onto the reference genome, iron-replete and -deplete samples were compared to control (input DNA), and signal-to-noise (S/N) ratio for peak calling was generated. Fur specifically binds various genomic loci under both conditions, but most of the ChIP peaks showed higher binding sites under iron-replete condition. Arrows indicate regions where ChIP peaks are associated with iron regulation.

Using computational prediction, PRODORIC (Münch et al., 2005), 26 different pathways involved in respiration, metabolism, and virulence were identified as likely regulated by *MAP3773c* (Figure 1). To confirm the findings from the *in silico* analysis and determine which genes are regulated by Fur in *MAP*, chromatin immunoprecipitation followed by deep sequencing (ChIP-seq) was performed. A custom-synthesized anti-Fur antibody capable of detecting the *MAP* Fur protein in its native form in *MAP* K-10 (Figure 2C) was used to generate ChIP binding profiles for *MAP* K-10 cultured under iron-replete and -deplete conditions (Figure 3).

ChIP peaks were called when the sequence coverage of genomic regions in the different treatments is enriched when compared to ChIP-seq control sample where the immunoprecipitation step was omitted (Strino and Lappe, 2016). Input DNA (no ChIP enrichment) had 34,907,295 (79.02% coverage against the *MAP* K-10 genome) uniquely mapped reads while ChIP-enriched DNA from iron-replete and iron-deplete conditions had 22,566,602 (55.66%) and 4,299,792 (14.73%) mapped reads, respectively.

Applying a *P*-value at ≤ 0.001, the ChIP-seq assay identified nine Fur binding sites out of 14 previously predicted by PRODORIC. ChIP-seq analysis revealed a total of 5,381 and 4,960 binding sites of Fur protein in the *MAP* K-10 genome (signal-to-noise ratio) under iron-replete and iron-deplete conditions, respectively (Figure 3).

**FIGURE 4:**
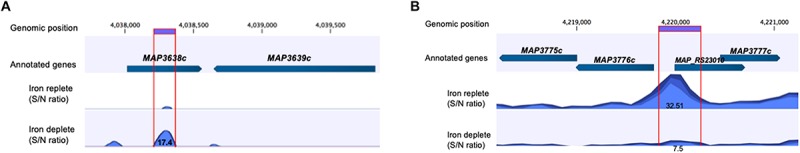
Applying FDR ≤ 10^– 50^, there are three ChIP peaks associated with iron regulation. **(A)** *MAP* Fur protein binds to the region of *MAP3638c*; however, only under iron-deplete condition is binding statically significant with a peak score of 17.4 (*MAP3638c*). **(B)** Under iron-replete conditions, there is a strong binding of MAP Fur to the region of *MAP3776c* represented by a peak score of 32.51.

Applying a FDR at ≤ 10^–50^, under iron-replete conditions, a total of 43 significantly enriched regions were identified on the K-10 genome (Table 1). Peaks were either localized between open reading frames (ORFs) (27%; intergenic regions) and within annotated genes (73%). In contrast, under chelation treatment (iron depletion), 11 enriched regions were identified (Table 2), all showing binding sites within ORFs. Four ChIP peaks were present under both iron-replete and -deplete conditions simultaneously (Table 3). Diverse functions are encoded by genes where Fur bound on the *MAP* K-10 genome: cell wall synthesis, energy metabolism, respiration, and transcriptional/translation regulation. Out of 58 genes (FDR ≤ 10^–50^) from both conditions (Tables 1–3), 11 are annotated as hypothetical proteins, 2 are described as pseudogenes (Table 4), and three ChIP peaks are associated with iron regulation: *MAP3638c*, *MAP3736c*, and *MAP3776c.* Interestingly, Fur bound upstream of *MAP3776c*, an ABC transporter, only under iron-replete condition and binding to *MAP3638c* (hemophore-like protein) was identified only under iron-deplete conditions (Figures 4A,B).

**TABLE 1:** List of genes regulated by Fur under iron-replete conditions, FDR ≤ 10^–50^.

**Gene**	**Peak score**	**FDR.bonf**	**Function**	***P*-value**
MAP3776c	32.51	4.18E-228	ABC transporter ATP-binding protein	4.04E-232
MAP1134	23.71	1.61E-120	16S rRNA m5C967 methyltransferase	1.55E-124
MAP4122	22.29	2.41E-106	Hypothetical protein	2.33E-110
MAP2627c	22.09	2.16E-104	Hypothetical protein	2.09E-108
MAP1398	21.41	5.37E-98	Hypothetical protein	5.19E-102
MAP1129	20.53	5.64E-90	Lysoplasmalogenase	5.46E-94
MAP3230c	20.5	1.04E-89	AraC family transcriptional regulator	1.00E-93
rpmG	19.86	4.49E-84	50S ribosomal protein L33	4.34E-88
MAP2419	19.61	7.04E-82	Membrane protein	6.81E-86
MAP2389c	19.59	1.03E-81	Amidohydrolase	9.96E-86
aroA	19.51	4.62E-81	3-Phosphoshikimate 1-carboxyvinyltransferase	4.47E-85
MAP2370c	19.25	7.14E-79	Short-chain dehydrogenase	6.90E-83
MAP2011	19.23	1.12E-78	Hypothetical protein	1.08E-82
MAP0130	19.2	1.93E-78	ATP-binding protein	1.87E-82
MAP2640c	18.84	1.86E-75	CPBP family intramembrane metalloprotease	1.80E-79
MAP2620c	18.73	1.56E-74	Nitrate reductase subunit alpha	1.51E-78
MAP1360	18.7	2.47E-74	Phenylalanine–tRNA ligase subunit beta	2.39E-78
MAP2969c	18.65	6.83E-74	Hypothetical protein	6.61E-78
MAP0351	18.64	7.32E-74	Transcriptional regulator	7.07E-78
MAP3430	18.53	6.66E-73	Phosphomannomutase	6.44E-77
MAP2173c	18.46	2.42E-72	Pseudo	2.34E-76
MAP2465c	18.36	1.50E-71	Hypothetical protein	1.45E-75
MAP0636	18.19	3.12E-70	CPBP family intramembrane metalloprotease	3.02E-74
MAP2744c	17.74	1.03E-66	Catalase-related peroxidase	9.97E-71
MAP0867c	17.61	9.82E-66	LLM class F420-dependent oxidoreductase	9.50E-70
rsmD	17.59	1.40E-65	16S rRNA (guanine(966)-N(2))-methyltransferase RsmD	1.35E-69
MAP_RS19330	17.47	1.27E-64	ANTAR domain-containing protein	1.22E-68
MAP2411	17.36	8.04E-64	Pyridoxamine 5′-phosphate oxidase	7.77E-68
MAP0357	17.28	3.43E-63	Membrane protein	3.32E-67
MAP2395c	17.13	4.66E-62	Enoyl-CoA hydratase/isomerase family protein	4.51E-66
MAP2479	17.1	7.40E-62	Potassium transporter TrkA	7.16E-66
rsgA	17.1	8.08E-62	Ribosome small subunit-dependent GTPase A	7.81E-66
MAP3477	16.78	1.83E-59	Pseudo	1.77E-63
MAP2747	16.72	4.92E-59	Long-chain-fatty-acid–CoA ligase	4.76E-63
MAP3486	16.49	2.23E-57	Lactate 2-monooxygenase	2.15E-61
MAP1560	16.49	2.40E-57	Esterase	2.32E-61
MAP3063	16.45	4.56E-57	1,4-Alpha-glucan-branching protein	4.41E-61
MAP3015	16.33	3.20E-56	Short-chain dehydrogenase/reductase	3.10E-60
MAP1161	16.09	1.54E-54	Hypothetical protein	1.49E-58
DkgA	15.88	4.35E-53	2,5-Diketo-D-gluconic acid reductase	4.21E-57
MAP0988	15.82	1.18E-52	Nucleoside triphosphate pyrophosphohydrolase	1.14E-56
MAP1227	15.56	6.84E-51	Methylmalonyl Co-A mutase-associated GTPase MeaB	6.61E-55
MAP3488c	15.53	1.08E-50	Hypothetical protein	1.04E-54

**TABLE 2:** List of genes regulated by Fur under iron-deplete conditions, FDR ≤ 10^–50^.

**Gene**	**Peak score**	**FDR.bonf**	**Function**	***P*-value**
MAP_RS12480	19.48	7.85E-81	23S ribosomal RNA	7.59E-85
MAP3664	18.75	1.04E-74	Glycosyl transferase	1.01E-78
Rrf	18.04	4.50E-69	5S ribosomal RNA	4.35E-73
MAP3638	17.74	1.03E-66	Hemophore	9.92E-71
MAP0182c	16.39	1.25E-56	Hypothetical protein	1.20E-60
MAP2957	16.15	6.05E-55	Peptidase M23	5.85E-59
MAP_RS12480	16.11	1.17E-54	23S ribosomal RNA	1.13E-58
MAP3471c	15.79	1.76E-52	Hypothetical protein	1.70E-56
MAP_RS14585	15.51	1.61E-50	Hypothetical protein	1.56E-54
MAP2961c	15.5	1.68E-50	DNA-protecting protein DprA	1.63E-54
MAP1420	15.44	4.45E-50	Non-ribosomal peptide synthetase	4.30E-54

**TABLE 3:** List of genes regulated by Fur under iron-replete and -deplete conditions, FDR ≤ 10^–50^.

		**Peak score**		**FDR. Bonferroni**	
		**Replete**	**Deplete**	**Replete**	**Deplete**
Gene function	MAP3736c ABC transporter ATP-binding protein	38.74	33.46	0.00	9.02E-242
	MAP2381 acetoin dehydrogenase	26.50	26.02	5.07E-151	1.70E-145
	MAP2071c cyclohexanecarboxylate-CoA ligase	17.59	21.33	1.49E-65	3.10E-97
	MAP2840c diaminopimelate epimerase	19.99	16.79	3.42E-85	1.39E-59

**TABLE 4:** List of genes regulated by Fur under iron-replete (yellow) and iron-deplete (gray) conditions with no function assigned, ≤ 10^–50^.

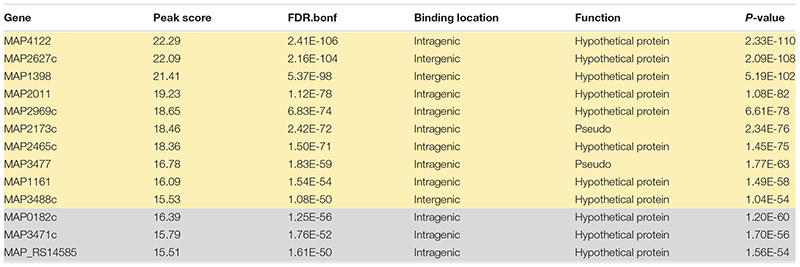

### Fur Binds to Fur Box Motif Under Iron-Replete or -Deplete Condition

**FIGURE 5:**
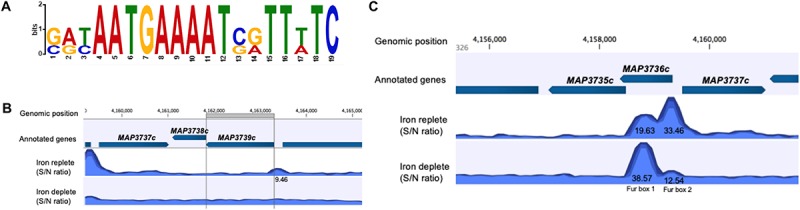
*MAP* Fur box analysis. **(A)** The most significant motif derived from ChIP-seq binding sequence using MEME. Height of each letter represents the relative frequency of each base at a different position in the consensus sequence. **(B,C)** A zoom-in of the *MAP* Fur boxes generated by CLC genomics. **(B)** Under both iron conditions, there is no binding of *MAP* Fur to the region of Fur box 3 (*MAP3739c*). ChIP peak (9.46) outside the ORF has FDR higher than the threshold of FDR ≤ 10^– 50^ **(C)** The enriched region of *MAP* Fur binding onto Fur Box 1 and 2 identified by ChIP-seq. ChIP peak showed higher occupancy under iron-deplete condition in the Fur Box 1 region. S/N denotes the signal-to-noise ratio for peak calling generated by CLC software.

Fur box consensus sequence was identified in ChIP-seq data using MEME-ChIP (Figure 5A). FIMO (Find Individual Motif Occurrences) analysis identified 15 occurrences of Fur box motif (*P* ≤ 0.001), 12 of them presented under iron-replete conditions and 3 under iron-deplete condition (Table 5).

**TABLE 5:** FIMO output. Most significant Fur box motif (SRYAATGAAAAT SRTTWTC) derived from ChIP-seq binding in iron-replete (yellow) and -deplete (gray) conditions.

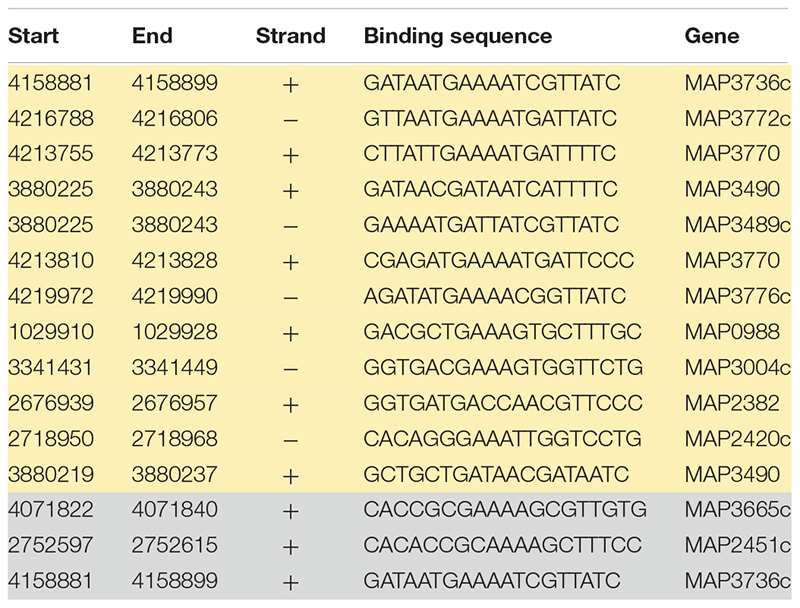

From previous studies, it is known that the *MAP* K-10 genome contains three Fur box motifs (Stratmann et al., 2004). However, data from ChIP-seq showed that the Fur protein does not show significant binding (FDR ≥ 10^–50^) to the region of Fur box 3 (*MAP3739c*) (Figure 5B). The highest peak score from all ChIP-seq data was observed within and just upstream of *MAP3736c*, located on LSP14, *MAP*-specific genomic island (Alexander et al., 2009). Within *MAP3736c* (located between nucleotides 4158368 and 4159327), there are two putative Fur Boxes: Fur box 1 (located between nt 4158681 and 4158966 of the genome) and Fur box 2 (located between nt 4159132 and 4159456) (Stratmann et al., 2004). ChIP-seq analysis showed high binding in both regions, confirming the exact location (Figure 5C). When intracellular Fe^2+^ was depleted by the addition of 2,2-dipyridyl, *MAP* Fur bound with higher affinity to Fur box 1 region (peak score = 38.57) in contrast to a lower binding score for Fur box 2 (peak score = 12.54), while under replete conditions, where *MAP* was grown in complete media, the opposite was observed, a lower *MAP* Fur binding in the Fur box 1 region (peak score = 19.63) and a higher peak in Fur box 2 region (peak score = 33.46).

### Validation of MAP Fur Binding

To confirm binding to the Fur promoter region, biotinylated or unlabeled PCR fragment including Fur box 1q identified by ChIP-seq was amplified and subjected to an electrophoretic mobility shift assay (EMSA) using purified *MAP* Fur protein (Figures 2A,B).

**FIGURE 6:**
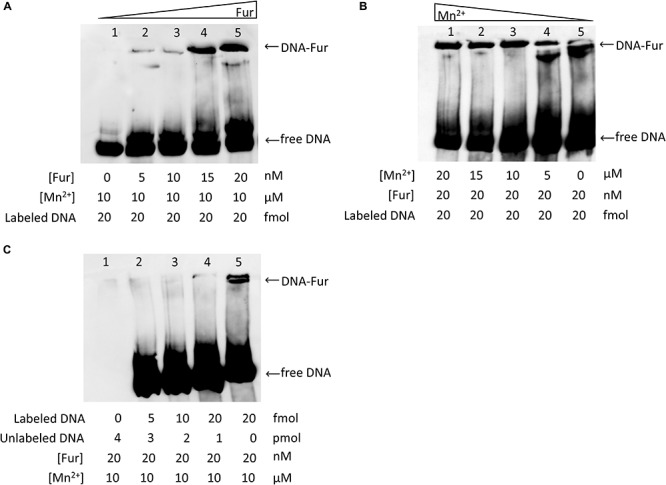
EMSA analysis of *MAP* Fur binding to Fur box consensus DNA. Binding activity is represented by band intensity. Twenty femtomoles of *MAP* DNA including the Fur Box 1 consensus biotin labeled was run in a 5% native polyacrylamide gel with different concentrations of MAP Fur protein and Mn^2+^. **(A)** Protein–DNA binding is dose-dependent: titration of purified *MAP* Fur protein shows an increase of binding activity as more protein is added to the system. **(B)** Binding activity is more efficient in the presence of Mn^2+^: No addition of Mn^2+^ (Lane 5) binding occurs with a lower band intensity when compared to the sample with Mn^2+^ (Lanes 1–4). **(C)** Competitive EMSA. Fur protein was incubated with either biotin-labeled DNA probe or unlabeled DNA probe or with both. Biotin-labeled probe was detected using chemiluminescence-based nucleic acid detection kit. Addition of unlabeled DNA affects binding activity, showing binding specificity.

Titration of Fur protein in the presence of Mn^2+^ and 20 fmol of DNA showed that binding is dose-dependent, as the Fur concentration was increased, there was an increase of binding activity (Figure 6A). However, in the absence of Mn^2+^, Fur binding to DNA was not as efficient as in the presence of Mn^2+^ (Figure 6B).

Furthermore, DNA–protein complex was specific to Fur binding site, as shown in the competition assay (Figure 6C), and adding a different concentration of excess unlabeled Fur box 1 probe competed with and abrogated labeled Fur box 1 probe binding to Fur protein.

## Discussion

In this study, a full characterization of the Fur in *MAP* was performed. Fur and its involvement in iron homeostasis are well known in bacteria such as *E. coli*, *Bacillus subtilis*, and *Salmonella Typhimurium.* This protein has been shown to work as a repressor, by blocking RNA polymerase binding to the promoter region of genes involved in iron homeostasis by repressing transcription (Escolar et al., 1997), but can also work as an activator by positively regulating gene expression in response to iron through indirect mechanism involving repression of small regulatory RNA (Delany et al., 2001; Masse et al., 2005). The current study confirmed, by Western blot (Figure 2A) and mass spectrometry (Figure 2B), that *MAP3773c* encodes a Fur-like protein in *MAP.* A regulatory network of *MAP* Fur binding sites was identified using three independent approaches: (1) *in silico* (PRODORIC), (2) *in vivo* (ChIP-seq), and (3) *in vitro* (EMSA). *In vivo* and *in vitro* analyses established that Fur binding was responsive to iron availability.

ChIP-seq analysis expanded the number of *MAP* Fur binding sites, from 14 genes predicted by PRODORIC to 58 enriched binding regions (FDR ≤ 10^–50^). Binding locations were distributed almost evenly between intragenic and intergenic regions. While binding of Fur in intragenic regions refute the definition of a transcriptional factor (Browning and Busby, 2004), recent ChIP-seq studies with *M. tuberculosis*, *E. coli*, *Salmonella*, and *Corynebacterium* reported intragenic TF binding that play critical roles in transcription and significantly affect regulation of gene expression (Dillon et al., 2012; Fitzgerald et al., 2014; Knapp et al., 2015). Additionally, during characterization of the Fur regulon in *Pseudomonas syringae*, Butcher et al. (2011) did not observe general differences between Fur binding to intergenic and intragenic sites. Both showed comparable binding affinity in *P. syringae*, suggesting that, although 100% of *MAP* Fur binding under iron-deplete conditions are located in intragenic regions, *MAP* Fur can be biologically active and able to bind specific DNA sequences to control gene expression.

Iron regulation by Fur in *MAP* appears to be more complex than the classic model, where Fur acts as a repressor when sensing high intracellular Fe^2+^. It then forms the Fur–Fe^2+^ complex and binds to the Fur box sequence, which enables Fur transition from its inactive (*apo*-) to its activated (*holo*-) form (Hantke, 2001; Helmann, 2014). Additionally, data from the present study showed that *MAP* uses Fur in the absence of intracellular Fe^2+^, a process known as *apo*-regulation. In low-iron conditions, *apo*-Fur protein binds to the promoters of its target genes and regulates transcription (Miles et al., 2010).

The complexity of Fur regulation can be exemplified in the ChIP peak of *MAP3736c*, where *apo*-Fur binds to Fur box 1 under iron-deplete conditions and *holo*-Fur binds to Fur box 1 and 2 under iron-replete condition. The physiological significance of *apo*-Fur binding in *MAP* is unclear, however, previous studies with *Helicobacter pylori* showed that when iron levels are low, genes responsible for iron storage are repressed by *apo*-Fur (Bereswill et al., 2000). Furthermore, additional studies in *Campylobacter jejuni* showed that expression genes controlled by Fur was decreased in the wild-type strain under iron-deplete condition and, in a Fur knockout strain, expression was increased (Holmes et al., 2005), indicating that *apo-*Fur plays an important role in iron metabolism. Corroborating this result, ChIP-seq analysis identified *apo*-Fur binding to *MAP3638c*, only under iron starvation. MAP3638c is a hemophore-like protein, suggesting that *MAP* likely uses heme as an additional iron source as previously described in *M. tuberculosis* (Tullius et al., 2011).

Finally, to confirm and validate Fur-Fur box1 binding, an EMSA using PCR amplification of ChIP-seq-identified Fur box 1 and purified Fur-like protein (*MAP3773c)* was performed. The binding was dependent on the availability of Mn^2+^, a common surrogate metal that, unlike Fe^2+^, is stable in the presence of oxygen but promotes DNA binding and adopts the same coordination geometry as Fe^2+^ (Butcher et al., 2011). Additionally, a competitive gel shift assay confirmed specificity of *MAP* Fur binding to the Fur box 1 region. Taken together, the identification of consensus Fur box by ChIP-seq peaks combined with data from EMSA confirms that iron regulation in *MAP* is also mediated by a Fur homolog that recognizes the 19-bp DNA sequence, known as Fur box.

In this current study, we were not able to confirm Fur box 3 (*MAP3739c*) region as binding site for Fur protein as described by Stratmann et al. (2004). Computational methods as used by the group predicted binding sites relying on data available 15 years ago, which was likely incomplete. Further, most computational predictions of TF binding are prone to false discovery and need to be validated (Karimzadeh and Hoffman, 2018). By using directly and quantitatively sequencing in combination with specific antibody, as used in this currently study, ChIP-seq method provides a powerful strategy for identifying *in vivo* binding sites across entire genome (Collas, 2019).

## Concluding Remark and Future Directions

**FIGURE 7:**
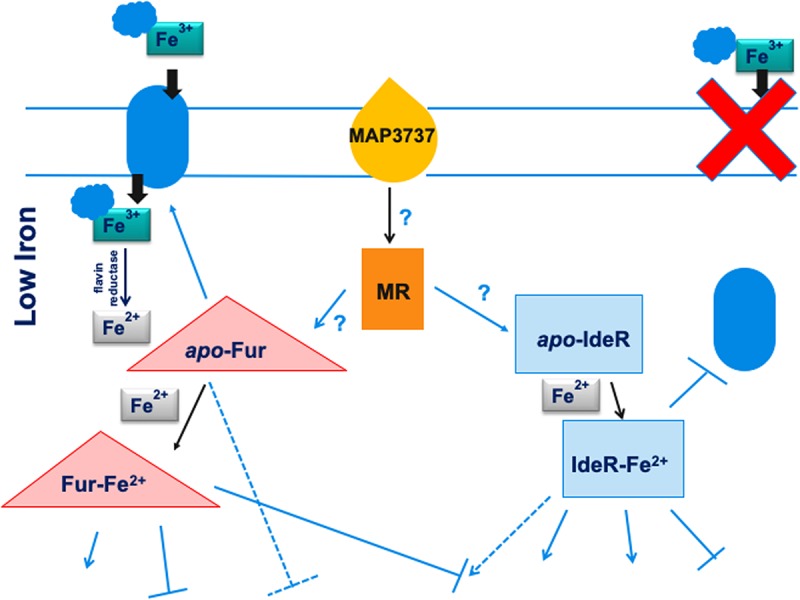
Model for the regulation of the iron stimulon in *M. paratuberculosis.* Under low-iron conditions, the iron sensor *MAP3737* (yellow teardrop) initiates a signal transduction cascade activating a hypothetical master regulator (MR) of the stimulon (orange rectangle) leading to the transcription of *apo*-Fur and *apo*-IdeR. Under low iron, *apo*-Fur activates transcription of the iron uptake protein or system (blue oval) that is transported to the cell membrane and carries ferric iron bound to carboxymycobactin (cMyco) (blue cloud) into the bacterium. The cMyco + Fe^3+^ complex possesses FAD-binding activity, allowing interaction with an iron flavin reductase that converts Fe^3+^ to Fe^2+^ and disassociates the complex, liberating Fe^2+^. *Apo*-IdeR is inactive but, bound to iron (IdeR Fe^2^), represses transcription of iron import/export protein or system and iron transport is shut down (red X). In addition, bound to ferrous iron, either regulator can exert a positive (pointed blue arrows) or negative regulation (flat-headed arrows) on the transcription of genes in their corresponding regulons. Apo-Fur also exerts a regulatory effect on the Fur regulon. Some genes may be controlled by both Fur and IdeR in opposite ways (broken blue arrows). More speculative effects are depicted by arrows with question marks. Thus, in this model, both Fur and IdeR act in a coordinate fashion to regulate the iron stimulon composed of the Fur and IdeR regulons. Black pointed arrows are used for processes unrelated to transcription such as binding or signal transduction effects.

In this work, we characterized *MAP3773c*, the Fur in *MAP*, using ChIP-seq. A genomic view of the *MAP* Fur regulatory network was identified, and several putative binding sites involved during iron-replete and -deplete conditions were discovered. Although this study is not a full description of the Fur regulon, our findings indicate that *MAP* Fur is a global regulator that recognizes many target sites in the genome, either by *apo*- or *holo*-Fur. Based on the proposed model by Lamont et al. (2013) where, in response to nitric oxide stress, *MAP3737* (PPE family protein) acts as the iron sensor protein and promotes expression of *MAP3734c-3736c*, leading to activation of the iron uptake system, we hypothesize a stimulon regulatory pathway with two regulatory proteins (Fur and IdeR). In *M. tuberculosis*, genes from the PPE family are upregulated during iron limitation and are repressed by IdeR, suggesting possible involvement of *MAP3737* in iron metabolism (Rodriguez et al., 2002). Thus, we proposed (Figure 7) that, during low-iron conditions, the iron sensor protein (*MAP3737*) activates a hypothetical master regulator (MR). The activation signal, which may or may not involve a phosphorylation cascade, leads to the transcription of *apo*-Fur that subsequently activates transcription of the iron uptake system. This leads to transport of carboxymycobactin (cMyco) into *MAP*. The cMyco + Fe^3+^ complex possesses FAD-binding activity, allowing it to interact with and activate the flavin iron reductase reducing Fe^3+^ to Fe^2+^. This is followed by disassociation of iron from the cMyco + Fe^3+^ complex and subsequent binding of liberated Fe^2+^ by Fur and IdeR. Fur–Fe^2+^ and Ide–Fe^2+^ can exert positive or negative regulation on the transcription of genes in their corresponding regulons. Further analysis of the complete *MAP* Fur regulon is underway; combining ChIP-seq data analysis from this work with another genome-scale experiment will provide a full understanding of direct or indirect roles of Fur in response to iron availability. To have a complete understanding of the *MAP* iron stimulon model, future studies will involve a basic understanding of Fur–IdeR interactions and how one or the other may be functional in *MAP* under a variety of *in vivo* and environmental conditions.

## Data Availability Statement

All datasets generated for this study are included in the article/supplementary material.

## Author Contributions

SS conceived the idea, obtained funding, helped to develop a study design, and edited the manuscript. JB helped to develop the study design, served as a co-investigator on the USDA grant, developed the recombinant protein, and edited the manuscript. RB helped in the study design, served as a co-investigator on the grant, developed Figure 7 (a model for iron stimulon in MAP), and edited the manuscript. TJ analyzed the ChIP-seq data. FS developed the study design, performed all experiments, analyzed the data, and wrote the manuscript.

## Conflict of Interest

The authors declare that the research was conducted in the absence of any commercial or financial relationships that could be construed as a potential conflict of interest.
